# Admission biomarkers and COVID-19 mortality: A retrospective study during Vietnam’s pandemic peak

**DOI:** 10.62838/jccm-2026-0003

**Published:** 2026-04-30

**Authors:** Uyen Thi Bich Nguyen, Hau Phuc Le, Vu Hoang Anh Nguyen, Ai-Thanh Tran-Nguyen

**Affiliations:** Department of Nephrology and Hemodialysis, Le Van Viet Hospital, Ho Chi Minh City, Vietnam; Outpatient Clinic for HIV-AIDS Patients, Chau Thanh District Medical Center, Tra Vinh Province,Vietnam; Can Tho University of Medicine and Pharmacy, Can Tho, Vietnam; Department of Psychosomatic Medicine, Thu Duc City Hospital, Ho Chi Minh City, Vietnam; Thu Duc City Hospital, Ho Chi Minh City, Vietnam

**Keywords:** COVID-19, prognostic biomarkers, mortality prediction, D-dimer, neutrophil counts

## Abstract

**Background:**

This study aimed to evaluate the prognostic value of key admission biomarkers in predicting mortality among hospitalized COVID-19 patients and to establish optimal cut-off thresholds for clinical decision-making.

**Methods:**

Retrospective cohort study included 269 COVID-19 patients treated at Thu Duc City Hospital, Vietnam, during the peak of the fourth pandemic wave in 2021. Logistic regression identified independent predictors of mortality, and receiver operating characteristic (ROC) curve analysis assessed the diagnostic performance of biomarkers. The area under the ROC curve (AUROC), Sensitivity, Specificity and Accuracy Index were used to determine optimal cut-off values.

**Results:**

Among the 269 patients, 53 (19.7%) died and 216 (80.3%) survived. Non-survivors exhibited elevated D-dimer (4.48 μg/mL vs 0.93 μg/mL, p < 0.0001), neutrophil counts (6.8 × 10^9^/L vs 3.5 × 10^9^/L, p < 0.0001) and white blood cell counts (11.68 × 10^9^/L vs. 7.87 × 10^9^/L, p < 0.0001). Lymphocyte counts and fibrinogen levels were significantly lower in non-survivors (p < 0.05). Logistic regression identified D-dimer (OR = 1.05, 95% CI: 1.02–1.09, p = 0.001), neutrophil counts (OR = 1.32, 95% CI: 1.10–1.63, p = 0.005) and lymphocyte counts (OR = 0.51, 95% CI: 0.26–0.92, p = 0.033) as significant predictors of mortality. ROC analysis revealed that D-dimer (AUROC = 0.809) and neutrophil counts (AUROC = 0.726) demonstrated strong discriminatory power, with cut-off values of ≥1.126 μg/mL (sensitivity = 90.57%, specificity = 60.19%) and ≥6.715 × 10^9^/L (sensitivity = 52.83%, specificity = 82.87%), respectively.

**Conclusion:**

These findings support the use of admission biomarkers to guide early interventions and improve patient outcomes in severe COVID-19 cases. Further studies are warranted to validate these results and explore their applicability in other settings.

## Introduction

The COVID-19 pandemic, caused by the novel coronavirus (SARS-CoV-2), primarily spreads through respiratory droplets and close contact with highly infectious individuals, posing significant challenges to global healthcare systems [1]. Although COVID-19 predominantly affects the lungs, and the majority of patients tend to have favorable outcomes, a subset experiences severe infections with rapid disease progression. This leads to coagulation disorders and ultimately, increased mortality [2, 3]. Consequently, the early identification of prognostic indicators is essential for guiding clinical diagnosis and optimizing treatment for COVID-19 patients. Three independent retrospective studies from Wuhan, China, collectively involving 1,392 patients, consistently revealed significant alterations in biochemical markers among those with poor outcomes. Specifically, these studies identified notable increases in D-dimer levels, prothrombin time, activated partial thromboplastin time (APTT), fibrinogen, white blood cell count, neutrophil count, blood urea nitrogen (BUN), creatinine concentration, and procalcitonin. Conversely, lymphocyte counts were significantly lower in deceased patients compared to survivors. Among these markers, elevated D-dimer levels emerged as a particularly strong predictor of mortality, alongside procalcitonin [4,5,6].

Two early studies conducted during the initial phase of the COVID-19 pandemic highlighted significant differences in hematological and biochemical markers between patients requiring ICU admission and those who did not. Wang et al. (2020) reported that ICU patients exhibited elevated leukocyte counts (1.5 times), neutrophil counts (1.5 times), and reduced lymphocyte counts (0.9 times). Additionally, levels of LDH (2.1 times), ALT (1.5 times), AST (1.8 times), total bilirubin (1.2 times), creatinine (1.1 times), cardiac troponin I (2.2 times), D-dimer (2.5 times), and procalcitonin (1.2 times) were significantly higher in ICU patients. The proportion of ICU patients with abnormal procalcitonin levels was also three times higher than those without ICU admission (75% vs. 22%; p < 0.001) [5]. Similarly, Huang et al. (2020) observed that ICU patients had elevated WBC counts (2.0 times), neutrophil counts (2.4 times), and reduced lymphocyte counts (0.4 times). Prothrombin time increased by 1.1 times, D-dimer levels by 4.8 times ALT and total bilirubin levels by 1.8 and 1.3 times, respectively, while AST levels rose 1.3 times. Albumin levels were lower (0.8 times), and elevated procalcitonin levels were seen in 25% of ICU patients compared to none in the non-ICU group (p = 0.029) [7].

Various biomarkers have demonstrated prognostic value in predicting mortality and severe progression in COVID-19 patients. Research has also shown that COVID-19 increases the risk of thrombosis, contributing to mortality and post-COVID complications. Biomarkers measured at hospital admission hold particular importance as they provide an early indication of disease severity and mortality risk, enabling timely clinical interventions [8]. While the pandemic has subsided, understanding the prognostic significance of these admission biomarkers remains crucial, not only for preparedness in future outbreaks of similar respiratory pathogens but also for managing post-COVID conditions and improving outcomes in other acute illnesses with comparable inflammatory profiles [9]. Therefore, this study aims to determine the cutoff values of key admission biomarkers to predict mortality, providing valuable insights to guide clinical decision-making and optimize patient management.

## Methods

### Study Context and Participants

This retrospective cohort study included 269 COVID-19 patients who were diagnosed via PCR and received inpatient treatment in the isolated COVID-19 treatment area of Thu Duc City Hospital, Ho Chi Minh City, between June and December 2021. This period coincided with the peak of the fourth COVID-19 wave in Vietnam, which overwhelmed the healthcare system, particularly in urban and suburban areas like Ho Chi Minh City [10]. Thu Duc City Hospital, a suburban facility with a 600-bed capacity, was among the designated COVID-19 treatment centers. The hospital established a dedicated and isolated inpatient ward to manage confirmed cases, implementing strict infection control measures in line with national guidelines to prevent cross-contamination.

The participants in this study were patients admitted to the isolated treatment area designated for COVID-19 management at Thu Duc City Hospital. These patients are presented with moderate to severe COVID-19, requiring hospitalization and specialized tiered care. Eligibility for inclusion was determined according to the five-tiered treatment model established by the COVID-19 Prevention and Control Steering Committee of Ho Chi Minh City. In this model, Tier 1 comprised asymptomatic or mild cases managed at isolation facilities or through home care; Tier 2 included patients treated at field hospitals; Tier 3 consisted of hospitalized patients with moderate disease requiring oxygen therapy; Tier 4 included severe cases managed at specialist hospitals; and Tier 5 represented critically ill patients requiring intensive care support [11].

Inclusion Criteria:

– Patients aged 18 years or older with a confirmed COVID-19 diagnosis by PCR.
– Patients with complete and accessible medical records.
– Patients classified as requiring inpatient care in tiers 2 to 5, in accordance with the five-tiered treatment model of the COVID-19 Prevention and Control Steering Committee.

Exclusion Criteria:

– Patients with pre-existing chronic hematologic diseases, chronic kidney disease, or cirrhosis.
– Patients with autoimmune diseases or those receiving chronic corticosteroid or other immunosuppressive therapies.
– Patients with a history of diagnosed psychiatric disorders that could interfere with the accuracy or reliability of data collection.

### Study Procedure

Patients meeting the inclusion criteria and not meeting any exclusion criteria were included in the analysis. Medical records of COVID-19 patients admitted to the hospital’s COVID-19 treatment area were reviewed. The research team extracted data specifically from the day of hospital admission, including consultation notes, clinical information, and relevant biomarkers. This data was used for analysis to evaluate the prognostic value of admission biomarkers in predicting patient outcomes.

### Statistical Analysis

Data analysis was conducted using R (version 4.3.1) for statistical computations and visualizations. Continuous variables were expressed as medians with interquartile ranges (IQRs), while categorical variables were presented as frequencies and percentages. Differences in continuous variables between survivor and non-survivor groups were assessed using the Wilcoxon rank-sum test, and associations in categorical variables were analyzed using the Chi-squared test, with statistical significance set at p < 0.05.

A logistic regression model was fitted to identify independent predictors of mortality. Odds ratios (ORs) with 95% confidence intervals (CIs) were computed to evaluate the strength of association between biomarkers and mortality. Multicollinearity was assessed using variance inflation factors (VIFs), with values > 5 indicating the presence of significant multicollinearity [12]. Biomarkers with significant associations were further analyzed to determine optimal cut-off thresholds.

Receiver Operating Characteristic (ROC) curve analysis was performed using the pROC package in R to assess the diagnostic performance of biomarkers. The area under the ROC curve (AUROC) was calculated for each biomarker to measure its ability to discriminate between survivors and non-survivors. Biomarkers with AUROC values ≥ 0.7 were deemed clinically significant [13]. Sensitivity, specificity, and accuracy metrics were used to establish these thresholds, balancing the trade-off between false-positive and false-negative rates.

ROC curves were generated using the roc() function from the **pROC** package. Separate ROC curves for each biomarker (e.g., D-dimer, Neutrophils, Lymphocytes, Hemoglobin, and Fibrinogen) were plotted on a single graph to visually compare their performance. The plot() function was used to overlay these curves, and a legend was added to distinguish between biomarkers.

### Ethical Considerations

The study was approved by Pham Ngoc Thach University of Medicine and Thu Duc City Hospital (IRB No.13/BV-HDDD). The research was conducted based on patient records without influencing treatment processes or patient benefits.

## Results

### Characteristics of participants

Among the 269 patients included in the study, in-hospital mortality was observed in 53 cases (19.7%), while 216 patients (80.3%) survived. Females accounted for a higher proportion of the cohort (58.4%). The mean age was 59.5 ± 16.7 years, and most patients were between 41 and 80 years of age, with the largest group (38.7%) aged 61–80 years. The mean BMI was 23.9 ± 3.5.

Hypertension was the most prevalent comorbidity (44.3%), followed by diabetes mellitus (20.5%), while respiratory diseases (2.6%) and cancer (1.9%) were uncommon. Regarding oxygen therapy at admission, most patients received oxygen via nasal cannula (53.2%), followed by invasive mechanical ventilation (21.6%), high-flow nasal cannula (18.2%), and face mask (7.1%) (Table 1).

**Table 1.** Patient Characteristics (n=269)

**Characteristics**		**Frequency**	**Percentage**	**Mean ± SD**
Gender	Male	112	41.6	
	Female	157	58.4	

Age group (years)	18 to 40	39	14.5	59.5 ± 16.7
	41 to 60	98	36.4	
	61 to 80	104	38.7	
	80 and above	28	10.4	

BMI (kg/m^2^)				23.9 ± 3.5

Comorbidity	Hypertension	119	44.3	
	Diabetes mellitus	55	20.5	
	Respiratory disease	7	2.6	
	Cancer	5	1.9	

Oxygen therapy	Nasal cannula	143	53.2	
	Invasive mechanical ventilation	58	21.6	
	HFNC	49	18.2	
	Mask	19	7.1	

Treatment Outcome	Mortality	53	19.7	
	Survival	216	80.3	

Statistical analysis revealed a significant association between age groups and treatment outcomes (p = 0.01). Mortality rates increased with age, with the highest proportions observed in patients aged 61 to 80 years (25.9%) and those over 80 years (28.6%), compared to significantly lower rates in younger groups (Table 2).

**Table 2.** Association of Age and Sex with Mortality in COVID-19 Patients

**Characteristics**	**Mortality (n=53)**	**Survival (n=216)**	p-value (*)
Sex			
Male	27 (24.1%)	85 (75.9%)	0.125
Female	26 (16.6%)	131 (83.4%)	

Age group			
Ages 18 to 40	2 (5.1%)	37 (94.9%)	**0.01**
Ages 41 to 60	16 (16.3%)	82 (83.7%)	
Ages 61 to 80	27 (25.9%)	77 (74.1%)	
Over 80	8 (28.6%)	20 (71.4%)	

Note: (*) Chi-square test

### Admission biomarkers in COVID-19 Patients and Association with Mortality

The analysis of admission biomarkers revealed significant differences between survivors and non-survivors among COVID-19 patients, highlighting the potential utility in predicting mortality. Non-survivors demonstrated lower platelet counts (196 × 10^9^/L vs 248.5 × 10^9^/L, p = 0.0076) and hemoglobin levels (12g/dL vs 13.8g/dL, p=0.026), suggesting hematologic disturbances as contributing factors to poor outcomes. Elevated WBC counts (11.68 × 10^9^/L vs 7.87 × 10^9^/L, p < 0.0001) and neutrophil counts (6.8 × 10^9^/L vs 3.5 × 10^9^/L, p < 0.0001) were prominent in non-survivors, reflecting a heightened inflammatory response. Conversely, non-survivors exhibited significantly lower lymphocyte counts (0.7 × 10^9^/L vs 0.92 × 10^9^/L, p = 0.0003), indicating immune suppression. AST and ALT also showed statistically significant differences between groups (both p = 0.01).

Among coagulation markers, D-dimer emerged as a strong predictor of mortality, with non-survivors showing markedly elevated levels (4.48 μg/mL vs 0.93 μg/mL, p < 0.0001), underscoring the role of hypercoagulability in adverse outcomes. Fibrinogen levels were also lower in non-survivors (2.5 g/L vs 3.1 g/L, p = 0.03), reflecting potential fibrinogen depletion in severe cases. Prothrombin time showed a minor but statistically significant difference between groups (13.2 seconds vs 14.1 seconds, p = 0.012), while activated partial thromboplastin time and creatinine levels did not significantly differ (Table 3).

**Table 3.** Admission Biomarkers in Survivors and Non-survivors with COVID-19

**Admission Biomarkers**	**Survival (n=216)**	**Mortality (n=53)**	**Both (n=269)**	p-value (*)
	**Median [25th–75th percentile]**			
**Complete Blood Count**				
Platelet Count (10^9^/L)	248.5 [182.5–334.5]	196 [147–277]	236 [171–318]	0.0076
White Blood Cells (10^9^/L)	7.87 [5.3–10.61]	11.68 [6.8–16.2]	8.19 [5.41–11.7]	<0.0001
Neutrophils (10^9^/L)	3.5 [2.6–5.8]	6.8 [3.5–12.1]	3.8 [3.1–6.5]	<0.0001
Lymphocytes (10^9^/L)	0.92 [0.63–1.35]	0.7 [0.41–0.98]	0.88 [0.59–1.28]	0.0003
Hemoglobin (g/dL)	13.8 [10.2–16]	12 [9–15.2]	13.8 [10.2–16]	0.026

**Biochemical Parameters**				
Creatinine (mg/dL)	0.67 [0.6–1]	0.67 [0.6–1]	0.67 [0.6–1]	0.624
AST (U/L)	29 [28–70]	28 [26–47]	29 [28–70]	0.01
ALT (U/L)	31 [30–72.5]	30 [28–50]	31 [29–72]	0.01

**Coagulation**				
D-dimer (μg/mL)	0.93 [0.47–1.86]	4.48 [1.58–9.20]	1.12 [0.55–3.79]	<0.0001
PT (seconds)	14.1 [12.9–14.7]	13.2 [12.9–14.6]	13.8 [12.9–14.7]	0.012
aPTT (seconds)	30.6 [26.7–33]	30.5 [28.3–33]	30.5 [26.7–33]	0.238
Fibrinogen (g/L)	3.1[2.12–3.8]	2.5 [1.81–3.8]	3.06 [2.12–3.8]	0.03

Note: (*) Wilcoxon test

### Prognostic Value of Biomarkers for Treatment Outcomes and Cut-off Thresholds

Multicollinearity assessment revealed severe collinearity between AST and ALT (VIF > 10); therefore, ALT was excluded from the final multivariable model. After refitting, VIF values for the remaining predictors ranged from 1.11 to 3.69, indicating no significant multicollinearity.

Logistic regression analysis identified several key biomarkers associated with mortality in COVID-19 patients. Significant predictors included increased D-dimer levels (OR = 1.05, 95% CI: 1.02–1.09, p = 0.001), neutrophil counts (OR = 1.32, 95% CI: 1.10–1.63, p = 0.005) and decreased lymphocyte counts (OR = 0.51, 95% CI: 0.26–0.92, p = 0.033), hemoglobin levels (OR = 0.85, 95% CI: 0.75–0.97, p = 0.015). Fibrinogen levels showed a borderline association with mortality, where lower fibrinogen levels trended towards higher risk (OR = 0.69, 95% CI: 0.48–0.99, p = 0.049). The model demonstrated a moderate fit (McFadden’s pseudo R^2^ = 0.320, p < 0.0001), suggesting that the included biomarkers are associated with mortality in this population.

These biomarkers reflect the complex interplay of inflammatory responses, coagulopathic disturbances, immune dysregulation, and hematologic alterations that contribute to adverse outcomes in severe COVID-19 cases. Notably, elevated D-dimer and neutrophil counts highlight the role of hypercoagulability and inflammation, while reduced lymphocyte and hemoglobin levels emphasize immune suppression and tissue oxygenation challenges as significant factors affecting mortality risk (Table 4).

**Table 4.** Logistic Regression Analysis of Admission Biomarkers Associated with Mortality in COVID-19 Patients

**Admission Biomarkers**	**OR**	**95% CI**	p-value (*)
D-dimer (μg/mL)	1.05	1.02 – 1.09	0.001
Creatinine (mg/dL)	0.95	0.22 – 4.05	0.946
AST (U/L)	1.00	0.98 – 1.01	0.551
WBC (10^9^/L)	1.04	0.90 – 1.18	0.598
Neutrophils (10^9^/L)	1.32	1.10 – 1.63	0.005
Lymphocytes (10^9^/L)	0.51	0.26 – 0.92	0.033
Platelets (10^9^/L)	1.00	0.99 – 1.00	0.253
Hemoglobin (g/dL)	0.85	0.75 – 0.97	0.015
PT (seconds)	0.93	0.75 – 1.13	0.468
aPTT (seconds)	0.95	0.86 – 1.05	0.317
Fibrinogen (g/L)	0.69	0.48 – 0.99	0.049

Note: (*) logistic regression analysis, report Odds Ratios

After performing logistic regression, the significant predictors identified were further evaluated using ROC curve analysis. The results demonstrate that D-dimer (AUROC = 0.809) and Neutrophil count (AUROC = 0.726) showed good discriminatory ability. These AUROC values exceed the commonly accepted threshold of 0.7, indicating meaningful predictive performance for mortality in this cohort.

**Fig 1.**
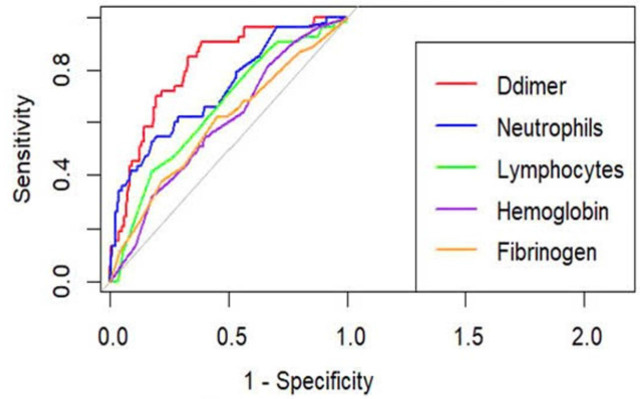
ROC Curves for D-dimer, Neutrophils, and Other Biomarkers in Predicting Mortality among COVID-19 Patients

In contrast, Lymphocyte count (AUROC = 0.655) shows moderate predictive ability but is less reliable than D-dimer and Neutrophils. Meanwhile, Fibrinogen (AUROC = 0.595) and Hemoglobin (AUROC = 0.598) have limited predictive value, indicating poor discrimination between survivors and non-survivors (Figure 1).

D-dimer: A cut-off value of ≥1.126 μg/mL was strongly associated with high mortality risk, achieving an AUROC of 0.809, sensitivity of 90.57%, and specificity of 60.19%, indicating a good balance between sensitivity and specificity. Additional cut-off values are presented in Table 5 to illustrate the relationship between D-dimer levels and diagnostic performance.

**Table 5.** Sensitivity, Specificity and Accuracy of D-Dimer Levels for Mortality Prediction in COVID-19 Patients

**Cut-off Threshold**	**Sensitivity**	**Specificity**	**Accuracy**
≥0.953 μg/ml	90.57%	50.00%	57.99%
≥1 μg/ml	90.57%	54.17%	61.34%
≥1.126 μg/ml	90.57%	60.19%	66.17%
≥1.67 μg/ml	73.58%	70.37%	71.00%

For neutrophils, a cut-off value of ≥6.715 × 10^9^/L showed high specificity (82.87%) but moderate sensitivity (52.83%), with an AUROC of 0.726, indicating acceptable discriminatory ability and potential utility for ruling in mortality risk in severe cases (Table 6).

**Table 6.** Sensitivity, Specificity, and Accuracy of Neutrophil Counts for Mortality Prediction in COVID-19 Patients

**Cut-off Threshold**	**Sensitivity**	**Specificity**	**Accuracy**
≥3.5165 ×10^9^/L	69.81%	51.85%	55.39%
≥4.3615 ×10^9^/L	66.04%	60.19%	61.34%
≥5.6 ×10^9^/L	60.38%	72.69%	70.26%
≥6.715 ×10^9^/L	52.83%	82.87%	76.95%

## Discussion

### D-dimer as the key prognostic marker

The median D-dimer level in our cohort (1.12 μg/mL) was higher than those reported in other studies, such as 0.8 μg/mL by Zhou et al. (2020) and 0.95 μg/mL by Kim et al. (2021) [14, 15]. This disparity could be explained by the higher mean age (59.5 years) and increased prevalence of comorbidities in our population, including hypertension (44.3%) and diabetes (20.5%).

In our study, non-survivors exhibited significantly higher D-dimer levels compared to survivors (median 4.48 μg/mL vs. 0.93 μg/mL, p < 0.0001), aligning with findings by Zhou et al. (2020) and Rodelo et al. (2012), which identified elevated D-dimer as a strong predictor of mortality [14, 16]. The AUROC for D-dimer in our analysis was 0.809, good discriminatory ability for mortality prediction. A threshold of ≥1.126 μg/mL provided high sensitivity (90.57%) but moderate specificity (60.19%), while a threshold of ≥1.67 μg/mL balanced sensitivity (73.58%) and specificity (70.37%).

Comparative studies have reported varying D-dimer thresholds based on population characteristics and clinical settings. Ganesan et al. (2021) proposed a threshold of >1.346 μg/mL with a sensitivity of 58.3% and specificity of 78.2%, emphasizing its prognostic value in severe cases requiring ICU admission [17]. Similarly, García-Cervera et al. (2021) identified a threshold of 1.2 μg/mL for venous thrombotic events, highlighting its utility in predicting complications beyond mortality [18]. Tang et al. (2020) suggested that D-dimer levels exceeding 3.0 μg/mL, six times the upper normal limit are associated with higher mortality risks but may also indicate a potential benefit from anticoagulant therapy, reducing mortality by approximately 20% [2, 19]. Furthermore, Soni et al. (2020) found that D-dimer levels ≥2.01 μg/mL were effective predictors of in-hospital mortality, particularly in patients with diabetes and advanced age [20].

These findings highlight the importance of tailoring D-dimer thresholds based on patient demographics and clinical context. Elevated D-dimer reflects a hypercoagulable state in severe COVID-19, potentially leading to venous thromboembolism, disseminated intravascular coagulation (DIC), and microvascular thrombosis, all of which contribute to higher mortality. Incorporating D-dimer monitoring into early risk stratification protocols and considering anticoagulant therapies at appropriate thresholds could enhance clinical outcomes in high-risk patients.

### Hematological Biomarkers and Inflammatory Response

Biochemically, we observed significant differences in AST and ALT levels between survivor and non-survivor groups at hospital admission, reflecting hepatic involvement in severe disease, potentially related to systemic inflammation, hypoxia, or drug exposure during treatment [21].

Non-survivors demonstrated significantly elevated neutrophil counts (median 6.8 × 10^9^/L vs 3.5 × 10^9^/L, p<0.0001) and reduced lymphocyte counts (median 0.7 × 10^9^/L vs 0.92 × 10^9^/L, p=0.0003). The AUROC for neutrophil counts was 0.726, with a cut-off value of ≥6.715 × 10^9^/L yielding a sensitivity of 52.83% and a specificity of 82.87%. These findings suggest that neutrophilia and lymphopenia are indicative of a dysregulated immune response, characterized by excessive inflammation and impaired viral clearance. Similar trends were observed in prior studies, where a high neutrophil-to-lymphocyte ratio (NLR) correlated with severe disease and poor outcomes. Inflammatory markers like neutrophils are often elevated due to cytokine release syndromes, while reduced lymphocyte counts reflect immune exhaustion and viral replication, both contributing to worse prognosis.

Additionally, while neutrophil counts showed a predictive AUROC of 0.726 in our study, Naoum et al. (2021) reported an AUROC of 0.744, highlighting consistency in the utility of this biomarker [22]. However, variations in thresholds across studies suggest the need for population-specific reference values.

Hypertension and diabetes mellitus were the most common comorbidities in our cohort, while respiratory diseases and malignancy were rare. These comorbidities may have influenced baseline biomarker levels and their dynamic behavior. Hypertension and diabetes are associated with chronic low-grade inflammation and endothelial dysfunction, which may predispose patients to elevated D-dimer levels and heightened inflammatory responses. Gogate et al. reported substantial overlap between COVID-19 related biomarkers and those implicated in common comorbid conditions, particularly metabolic disorders and malignancy, involving inflammatory and coagulation pathways such as D-dimer, neutrophil-related indices, and cytokine markers [23]. This overlap supports the notion that underlying comorbidities may amplify biomarker abnormalities observed in severe COVID-19 and partially confound their associations with mortality.

### Clinical Implications and Future Research

The identification of key biomarkers, including D-dimer, neutrophil counts, and lymphocyte counts, as significant prognostic indicators provide actionable insights for clinical practice. These biomarkers allow for the early identification of high-risk COVID-19 patients, facilitating timely interventions such as anticoagulation, immunomodulation, and enhanced monitoring. For example, the integration of D-dimer thresholds into clinical decision-making protocols could support stratified anticoagulation strategies to mitigate thrombotic complications, a common cause of mortality in severe cases. Similarly, tracking neutrophil and lymphocyte levels could aid in evaluating the inflammatory and immune response, enabling tailored treatment approaches. The practical thresholds established in this study, such as a D-dimer level ≥1.126 μg/mL and a neutrophil count ≥6.715 × 10^9^/L offer a foundation for risk stratification and targeted interventions. These findings align with global evidence, highlighting the utility of these biomarkers in guiding clinical management not only during the COVID-19 pandemic but also for similar respiratory pathogens in future pandemics.

Despite substantial advancements in understanding COVID-19 pathology and treatment strategies over the past four years, emerging variants continue to challenge the effectiveness of current preventive and therapeutic approaches. The dynamic nature of the virus has led to persistent issues, including delayed administration of antiviral therapies, diagnostic challenges with false-negative results, and inconsistent efficacy of some treatments. These factors, combined with the rapid progression of severe conditions such as ARDS, pulmonary embolism, disseminated intravascular coagulation, and cytokine storm, underscore the need for adaptive clinical strategies [24].

Future research should focus on validating these biomarkers across diverse populations, ensuring their generalizability and integration into standardized clinical algorithms. Additionally, the chronic consequences of COVID-19, including long COVID, warrant significant attention as they pose a growing healthcare challenge. Multidisciplinary approaches are needed to address these prolonged symptoms, which may require years of follow-up and management.

Emerging data presented at the 2024 Conference on Retroviruses and Opportunistic Infections emphasized the impact of hybrid immunity, stemming from prior infections and vaccinations on the progression and outcomes of SARS-CoV-2 infections. These findings reaffirm the continued importance of vaccination in preventing severe disease. However, challenges such as persistent RNA shedding in immunocompromised patients raise concerns about viral evolution and the potential emergence of more virulent strains [25].

### Strengths and Limitations

The study has several key limitations that should be noted. First, its retrospective design may introduce selection bias and limit the ability to establish causality between biomarkers and mortality. The reliance on pre-existing medical records may have also resulted in incomplete data collection. Second, the study was conducted at a single center during the peak of the COVID-19 pandemic in Vietnam, potentially limiting the generalizability of the findings to other settings or populations. Third, due to resource constraints during the pandemic, important biomarkers such as procalcitonin, interleukin-6 (IL-6), and lactate dehydrogenase (LDH) were not included in the analysis, which may have restricted the scope of the study. Finally, the lack of validated severity scores (e.g., APACHE II or SOFA), due to incomplete retrospective data, limited our ability to adjust for baseline disease severity in the analysis.

The study also has several strengths that enhance its significance. Conducted at an isolated COVID-19 treatment center during the peak of the pandemic in Vietnam, the research benefited from a well-structured healthcare environment with a highly skilled medical team managing patients. The comprehensive collection of admission biomarker data from moderate to severe COVID-19 cases provides valuable insights into disease progression and outcomes.

## Conclusion

This study demonstrates that admission biomarkers, particularly D-dimer, neutrophil count, and lymphocyte count, are independently associated with in-hospital mortality among hospitalized COVID-19 patients. These findings support the clinical utility of routinely available laboratory markers for early risk stratification. Despite its retrospective and single-center design, our study provides evidence to inform future prospective and multicenter research aimed at integrating biomarker-based assessment into clinical management of severe COVID-19.
